# Distinct [^18^F]FDG-PET imaging features of a newly recognized and yet uncharacterized RDD-ECD overlap disease entity

**DOI:** 10.1007/s00259-024-06751-5

**Published:** 2024-05-17

**Authors:** Martin W. Huellner, Marco M. Bühler, Viktor H. Kölzer, Perparim Limani, Wiebke Rösler

**Affiliations:** 1 Department of Nuclear Medicine, University Hospital Zurich, University of Zurich, Raemistrasse 100, Zurich, CH-8091 Switzerland; 2 Department of Pathology and Molecular Pathology, University Hospital Zurich, University of Zurich, Zurich, Switzerland; 3 Department of Surgery and Transplantation, University Hospital Zurich, University of Zurich, Zurich, Switzerland; 4 Department of Hematology and Oncology, University Hospital Zurich, University of Zurich, Zurich, Switzerland

## Abstract

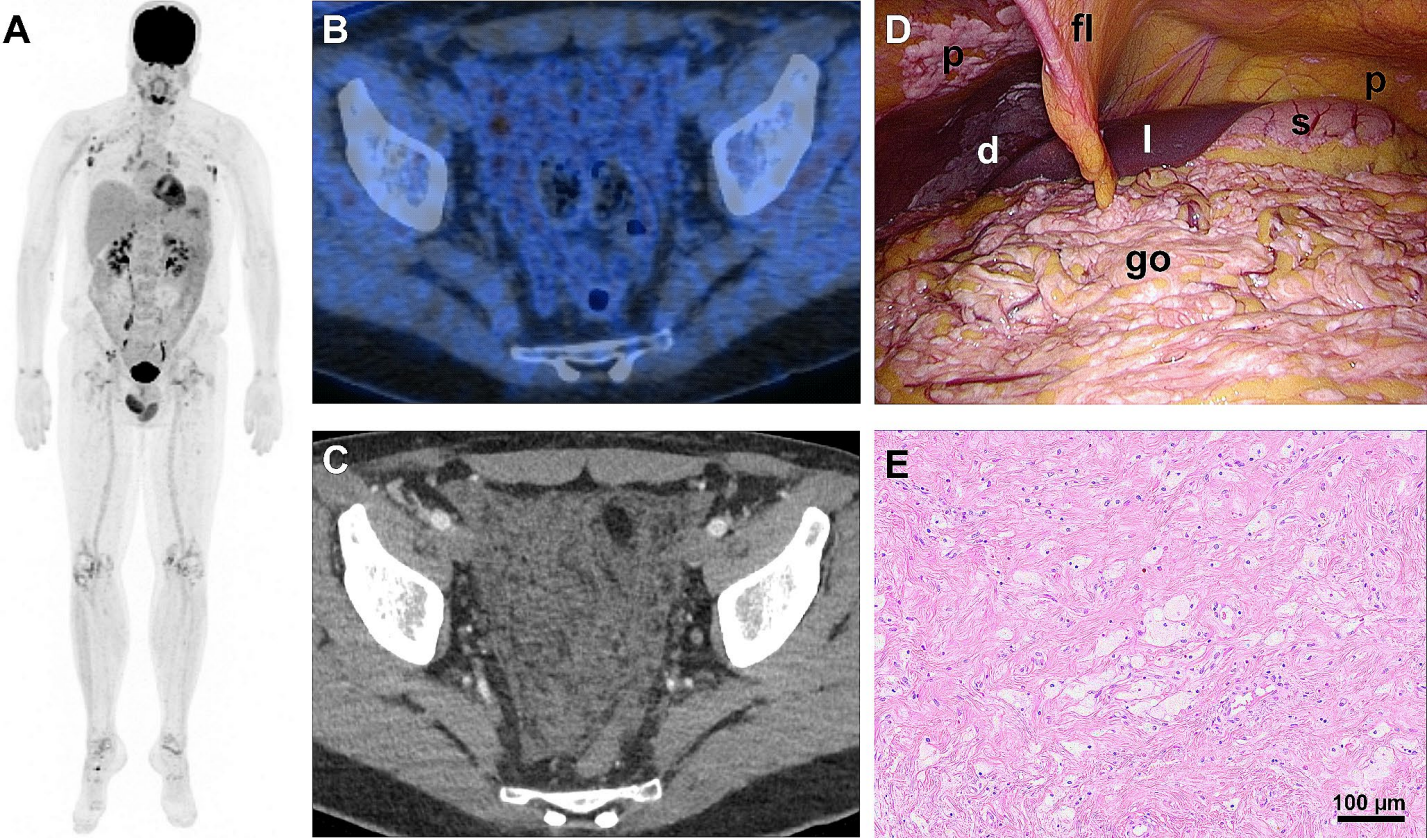

A newly recognized histiocytosis entity, encompassing clinical and histopathologic features of Rosai-Dorfman disease (RDD) and Erdheim-Chester disease (ECD), is driven by MAP2K1 mutations [1, 2]. [^18^F]fluorodeoxyglucose ([^18^F]FDG) positron emission tomography (PET) features have not yet been reported.

This 46 year-old man presented with a two-year history of clinical hallmarks resembling RDD rather than ECD, including lymphadenopathy and painless testicle enlargement [3], being also visible on [^18^F]FDG-PET (**A**). Testicular RDD-ECD involvement was also reported in 6/13 patients by Razanamahery et al. [2]. Diffuse omental proliferations, manifesting as faintly [^18^F]FDG-avid omental thickening resembling a fishing net (SUV_max_ 5.5; **A, B, C**), and symmetric large-joint synovitis were reported as specific features of RDD-ECD [1, 2], Notably, none of these features are characteristic of RDD or hitherto known ECD subtypes. Other RDD and/or ECD features were absent [4–7].

Open biopsy targeted peritoneal lesions (**D**) localized on the diaphragm (d), peritoneum (p) and greater omentum (go). Histopathology revealed nodular fibrosis, foamy cell infiltrates, pigment deposits and chronic perivascular inflammatory infiltrates (**E**). Molecular genetic analyses confirmed presence of a characteristic MAP2K1 mutation (p.Q56P).

Diamond et al. effectively treated a patient harboring the identical mutation with MEK inhibitors [8]. FAPI-PET focusing on fibrosis aspects of histiocytosis might help determining disease extent and assessing treatment response [9, 10].

In summary, the newly recognized RDD-ECD overlap histiocytosis demonstrates distinct [^18^F]FDG-PET features setting it apart from RDD and ECD. The concurrent presence of omental proliferations, symmetric large-joint synovitis, and high testicular uptake should raise suspicion for this yet uncharacterized disease.
